# Hyaluronan Synthesis, Catabolism, and Signaling in Neurodegenerative Diseases

**DOI:** 10.1155/2015/368584

**Published:** 2015-09-10

**Authors:** Larry S. Sherman, Steven Matsumoto, Weiping Su, Taasin Srivastava, Stephen A. Back

**Affiliations:** ^1^Division of Neuroscience, Oregon National Primate Research Center, Oregon Health & Science University, 505 NW 185th Avenue, Beaverton, OR 97006, USA; ^2^Department of Cell, Developmental & Cancer Biology, Oregon Health & Science University, 3181 SW Sam Jackson Park Road, Portland, OR 97239, USA; ^3^Department of Integrative Biosciences, School of Dentistry, Oregon Health & Science University, 3181 SW Sam Jackson Park Road, Portland, OR 97239, USA; ^4^Department of Pediatrics, Oregon Health & Science University, 3181 SW Sam Jackson Park Road, Portland, OR 97239, USA

## Abstract

The glycosaminoglycan hyaluronan (HA), a component of the extracellular matrix, has been implicated in regulating neural differentiation, survival, proliferation, migration, and cell signaling in the mammalian central nervous system (CNS). HA is found throughout the CNS as a constituent of proteoglycans, especially within perineuronal nets that have been implicated in regulating neuronal activity. HA is also found in the white matter where it is diffusely distributed around astrocytes and oligodendrocytes. Insults to the CNS lead to long-term elevation of HA within damaged tissues, which is linked at least in part to increased transcription of HA synthases. HA accumulation is often accompanied by elevated expression of at least some transmembrane HA receptors including CD44. Hyaluronidases that digest high molecular weight HA into smaller fragments are also elevated following CNS insults and can generate HA digestion products that have unique biological activities. A number of studies, for example, suggest that both the removal of high molecular weight HA and the accumulation of hyaluronidase-generated HA digestion products can impact CNS injuries through mechanisms that include the regulation of progenitor cell differentiation and proliferation. These studies, reviewed here, suggest that targeting HA synthesis, catabolism, and signaling are all potential strategies to promote CNS repair.

## 1. Introduction

Up to 20% of the volume of the mammalian central nervous system (CNS) is composed of an extracellular matrix (ECM) that includes proteins, proteoglycans, and glycosaminoglycans [1]. Evolving evidence indicates that the composition and organization of this matrix change throughout the course of normal aging, in neurodegenerative diseases and following CNS injury and that these alterations influence a wide range of cellular behaviors. The CNS ECM was originally believed to play mostly structural roles including supporting tissue architecture. Findings over the past few decades, however, indicate that the CNS ECM is an information-rich environment that includes signals that influence cell proliferation, differentiation, migration, synapse formation and remodeling, and responses to injury [2–7].

The composition and function of the CNS ECM can differ within distinct areas of the CNS [8]. For example, white matter ECM is far more diffuse compared to gray matter ECM. Within gray matter, perineuronal nets that surround some neuron cell bodies and dendrites are composed of a dense matrix of glycosaminoglycans, proteoglycans, tenascin R, and link proteins. This specialized ECM can regulate synaptic plasticity [9] while the negatively-charged glycosaminoglycans can influence the diffusion of cations that support rapid neuronal firing [10]. Another distinct ECM environment can be found in the basal lamina surrounding cerebral vessels, which is composed of collagen, fibronectin, perlecan, dystroglycan, and laminin-nidogen complexes. It surrounds the pial surface of the CNS and separates endothelial cells from the parenchyma, thus contributing to the blood-brain barrier [8].

Although numerous studies have implicated proteoglycans in the response to CNS damage and in mediating inflammatory responses and recovery (for excellent recent reviews see [11–13]) there is growing evidence that the glycosaminoglycan hyaluronan (HA) plays specific roles in regulating neural progenitor cell proliferation and differentiation. HA is a large unbranched, nonsulfated glycosaminoglycan composed of repeating disaccharide units of N-glucuronic acid and N-acetylglucosamine and is a major constituent of the ECM. HA can reach upwards of 25,000 disaccharide units, with molecular weights as high as 10^7^ Da. HA can have distinct physiological functions depending on its size, concentration, and localization. These functions include altering tissue hydration and elasticity and creating cell-free spaces that are crucial for cell migration. In addition, HA can induce cell signaling through transmembrane HA receptors.

HA is synthesized at the inner face of the plasma membrane and secreted into the extracellular space as a linear undecorated molecule. In mammals, HA synthesis is achieved by a family of transmembrane proteins known as HA synthases (HASs). The mammalian genome codes for three such synthases, HAS1, HAS2, and HAS3. HA catabolism is carried out by hyaluronidases (HYALs) that differ in their cellular localization and pH optima. Mammals possess multiple hyaluronidase genes, including HYAL-1 through HYAL-5, PH20/SPAM1, and, in humans, a pseudogene designated PHYAL-1. The balance between HA synthesis and catabolism has long been recognized to play roles in CNS development (e.g., [14–17]). Here, we review data implicating alterations in HA synthesis, signaling, and catabolism in the responses of progenitor cell populations to neurodegenerative diseases and CNS injuries and provide a framework to assess the efficacy of targeting HA as a strategy to promote CNS repair.

## 2. HA Accumulates in the Damaged CNS

### 2.1. Ischemic Injury

**Figure 1:**
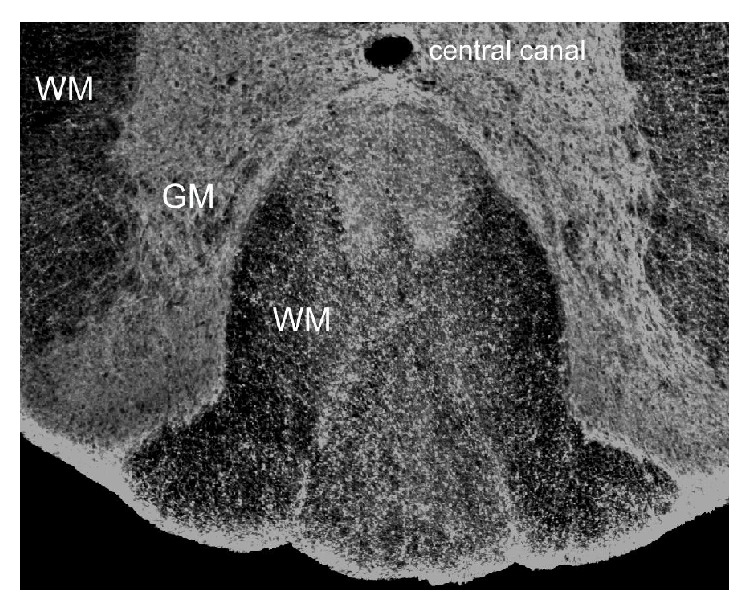
Distribution of HA (white) in lumbar spinal cord white matter (WM) and gray matter (GM). A section of a rat spinal cord was labeled with a biotinylated-HA-binding protein then visualized by fluorescence microscopy following staining with fluorescein-labeled streptavidin. Note that HA is diffusely distributed throughout white matter, but is much more dense in gray matter.

In the uninjured CNS, HA is diffusely distributed throughout the white matter but is densely packed in gray matter, including perineuronal nets (Figure 1). Damage to the CNS leads to HA accumulation [4, 18] in both white and gray matter that can persist for long periods of time after the initial insult. In most cases, pathological elevation of HA is linked to increased HAS transcription and is often associated with reactive astrogliosis and glial scarring. This effect has been most thoroughly studied in the context of ischemic injuries. For example, HAS2 mRNA is normally expressed at very low levels in the CNS but increased significantly within 6 hours following middle cerebral artery occlusion in rats [19]. HA was also elevated up to six weeks following a photothrombotic stroke lesion in adult mouse cortex [20]. Consistent with these experimental findings, HAS1 and HAS2 are elevated in infarcted and peri-infarcted tissues from patients following ischemic stroke [21]. Plasma levels of HA were also significantly elevated in acute stroke patients compared to controls and could predict 3-month functional outcomes, especially in patients with intracerebral hemorrhage [22].

In addition to the findings in adult patients and in adult models of stroke, HA is also markedly elevated in perinatal hypoxic-ischemic cerebral white matter injuries. Lesions resulting from these insults are the leading cause of cerebral palsy in survivors of premature birth and contribute to life-long neurobehavioral disabilities. HA accumulates in the ECM in human preterm chronic cerebral white matter lesions coincident with extensive reactive astrogliosis [23]. Similarly, in a fetal sheep model of cerebral hypoxic-ischemic injury, HA rapidly increased by 24 hours after a single episode of fetal hypoxia-ischemia and remained persistently elevated for weeks [24].

### 2.2. Seizures

Seizures also alter HA synthesis in the CNS but whether seizure activity directly increases or decreases HA is not entirely clear. Epileptic seizures can influence both the behaviors of existing neurons and adult neurogenesis (the generation of new neurons). One consequence of these alterations is abnormal sprouting by granule cell axons (so-called mossy fiber sprouting) in the dentate gyrus of the hippocampus, an area of the brain involved in learning and memory. Degradation of HA in organotypic hippocampal slice cultures with a hyaluronidase significantly reduced kainic acid-induced mossy fiber sprouting, a synaptic rearrangement associated with temporal lobe epilepsy [25]. Interestingly, HA and HAS3 both decrease in perineuronal nets in the hippocampus following seizures in a rodent model of temporal lobe epilepsy [26] while* Has3*-null mice have reduced hippocampal volumes and develop epileptic seizures [17]. These data suggest that reduced HA could contribute to seizure onset and recurrence but HA digestion by a hyaluronidase may generate HA products that reduce mossy fiber sprouting and, possibly, positive feedback circuits for seizure generation. In contrast to studies in rodents, HA was observed to increase in the hippocampus and temporal neocortex of patients with intractable mesial temporal lobe epilepsy [27]. It is possible that this discrepancy with the rodent studies is due to the fact that the human tissue samples had more chronic lesions with extensive astrogliosis compared to the rodent tissues and that HA was rebounding coincident with glial scarring.

### 2.3. Traumatic Brain and Spinal Cord Injuries

For up to 5 months after dorsal rhizotomy proximal to dorsal root ganglia, Mansour and coworkers [28] found increased glial hyaluronan-binding protein immunoreactivity (which typically reflects increased HA). In another study, spinal cord contusion injury in rats resulted in HA degradation at the site of injury with subsequent elevated accumulation within one month after injury [29]. HA was highest in areas where there was extensive astrogliosis. A study of controlled cortical impact injury in rats (as a model of traumatic brain injury) indicated that HAS1 and HAS2 mRNA increase anywhere from 4 hours to 3 days after injury compared to craniotomy alone [30]. This study did not, however, validate their findings by examining the distribution or levels of HA. Nonetheless, these data indicate that traumatic CNS injuries lead to long-term increases in HA in affected tissues.

### 2.4. Demyelinating Diseases

A number of studies have demonstrated increased HA in CNS lesions associated with neurodegenerative diseases that influence white matter. For example, HA is elevated in demyelinating white matter lesions from mice with experimental autoimmune encephalomyelitis and patients with multiple sclerosis, both of which are autoimmune diseases where myelin-reactive immune cells induce demyelination [31–33]. HA is also elevated in the brains of patients with Vanishing White Matter Disease, a genetic leukoencephalopathy caused by mutations in eukaryotic translation initiation factor 2B [34]. These patients experience a slowly progressive neurological deterioration with episodes of rapid clinical worsening triggered by stress. HA accumulation was especially pronounced in affected frontal white matter but not in the unaffected cerebellar white matter in these patients.

### 2.5. Normative Aging and Dementias

HA accumulation in the CNS also occurs during the course of normal aging. Moderate increases in HA were observed in 30-month-old rat brain tissue compared to tissues from younger animals [35]. HA levels also significantly increase with age in the gray matter of rhesus and Japanese macaques in areas with astrogliosis and coincident with increased transcription of* HAS1* expressed by reactive astrocytes [36]. HA is similarly elevated in gray matter from patients with Alzheimer's disease (AD) [37, 38] and the white matter of patients with vascular dementia who have a low burden of AD pathology accompanied by vascular injury consistent with vascular dementia [39]. In agreement with these findings, Nägga and coworkers [40] observed significantly increased levels of HA in the cerebrospinal fluid (CSF) from patients with vascular dementia but not AD alone compared with controls. This is consistent with the findings of Laurent and coworkers [18], who observed elevated HA in the cerebrospinal fluid (CSF) of patients with a number of different neurological diseases. Increased levels of HA were also observed in the CSF of individuals with vascular abnormalities determined as significant white matter changes or in patients with previous infarction compared with individuals without these findings [40]. There was a positive correlation between the levels of HA and the CSF/serum albumin ratio, an indicator of blood-brain barrier integrity, in patients with both vascular dementia and AD. These findings support the notion that HA accumulates in gray matter during the course of normal aging, and further increases in white matter in cases of age-related vascular injury.

All together, the findings described above demonstrate that HA accumulates, likely due to increased HAS transcription, in a wide variety of insults to the CNS. HA accumulation is usually associated with astrogliosis, and indeed reactive astrocytes demonstrate elevated HAS. This likely accounts for the chronically high levels of HA within glial scars. As indicated by studies in models of spinal cord injury and seizures, HA may initially become degraded due to the possible induction of specific hyaluronidases or other factors in the early injury environment but, at least in chronic lesions, quickly accumulates to higher than normal levels that are sustained for long periods after the initial insult. At least in some insults, this elevated HA can be detected in CSF. Future studies focused on examining when HA concentrations become detectably elevated in the CSF following different insults to the CNS will demonstrate the potential of HA as a diagnostic biomarker for CNS injury.

## 3. HA Accumulation Alters Neural Progenitor Cell Proliferation and Differentiation

### 3.1. Effects of HA on Neural Progenitor Cells and Glia

What are the consequences of prolonged HA accumulation in CNS lesions? In addition to its roles in nervous system development and homeostasis, HA can have an impact on nervous system repair by altering the proliferation, migration, and differentiation of progenitor cell populations in the CNS. In demyelinating lesions, including those of multiple sclerosis patients, some recovery of function is associated with the recruitment of oligodendrocyte progenitor cells (OPCs) that differentiate into oligodendrocytes (OLs) that remyelinate spared axons [41]. However, in chronic white matter lesions, OPCs accumulate and fail to give rise to myelinating OLs [42–47]. The presence of HA within demyelinating lesions correlates with areas where remyelination fails, and the addition of high molecular weight preparations of HA (>10^6^ Da) to rodent OPCs* in vitro*, to white matter slice cultures, or to lysolecithin-induced demyelinating corpus callosum lesions blocks OPC differentiation and remyelination [31, 32, 48]. Interestingly, high molecular weight HA also blocks astrocyte proliferation [29] and inhibits glial scarring [49]. Thus, although the accumulation of HA in demyelinating lesions is linked to the inhibition of OPC maturation and remyelination failure, it may also mediate glial scar formation.

HA may also play a role in OPC recruitment to demyelinating lesions. Following demyelinating insults, OPCs migrate towards damaged axons before differentiating into myelinating OLs. Using an OPC line, Piao and coworkers [50] found that reducing the expression of the HA receptor CD44 or treating cells with a CD44 blocking antibody inhibited OPC migration both* in vitro* and following transplantation into inflammatory demyelinating lesions. Although the authors reported elevated HA in these lesions, it remains to be determined if HA is directly required for the migratory behavior of these cells. Nonetheless, these data suggest that while HA can block OPC maturation when found in excess in demyelinating lesions, HA might also be required to facilitate OPC recruitment to lesions in a CD44-dependent manner.

### 3.2. Roles of HA in Neural Stem/Progenitor Cell Niches

While HA is mostly observed at low levels in the uninjured adult brain, it remains at high levels in the adult subventricular zone, the subgranular zone of the dentate gyrus of the hippocampus, and the rostral migratory stream, areas where neural stem/progenitor cells (NSPCs) reside throughout life [20, 51]. HA may, therefore, play a role in regulating NSPCs in their niches [52]. Although it is unclear what direct role HA plays in NSPC niches, a number of studies have examined the effects of HA gels on NSPCs and the use of these gels as vehicles in NSPC transplantation studies [52]. For example, HA hydrogels significantly improved the survival of a human NSPC line and glial-restricted precursor cells following transplantation into the brains of mice [53]. In another study, a biopolymer hydrogel composed of cross-linked HA and heparin sulfate significantly promoted the survival of NSPC lines* in vitro* in conditions of stress and* in vivo* following transplantation into the infarct cavity in a stroke model [54]. Degradation of the HA gels encapsulating neural progenitor cells caused differentiation and maturation of the progenitor cells.

Collectively, these studies suggest that HA within NSPC niches plays a critical role in regulating NSPC proliferation and differentiation. This function of HA is recapitulated in demyelinating and possibly other lesions, where OPCs encounter a niche-like environment rich in HA that can influence OPC migration, astrocyte proliferation, and glial cell differentiation. Presumably, the levels and distribution of HA within NSPC niches must be tightly controlled to regulate NSPC differentiation. The challenge will be to identify a means of controlling HA synthesis or catabolism within NSPC niches or areas of damaged CNS tissues in a way that promotes nervous system repair. The use of HA biomaterials, as described above, has the potential to regulate HA functions in both stem cell niches and CNS lesions, offering a potential approach to stem/progenitor cell-based therapies (reviewed in [52]).

## 4. Endogenous Hyaluronidases Influence CNS Repair

As discussed above, there are several mammalian hyaluronidases that catabolize HA in tissues. While digestion of high molecular weight HA can have its own biological consequences, the accumulation of HA digestion products generated by hyaluronidases can also have distinct, size-dependent activities.

### 4.1. Roles of Hyaluronidases in CNS Injury and Disease

The first indication that hyaluroidases play a role in the CNS came from the observation that there is a high level of hyaluronidase activity in the developing brain and spinal cord that declines during the early perinatal period [14]. Hyaluroindase activity and expression are generally low in the adult CNS. However, following a number of insults to the nervous system, hyaluronidase expression and activity increase. For example, in both stroke and peri-infarct regions of ischemic stroke patients, HYAL1 and HYAL2 were elevated in inflammatory cells, microvessels, and neurons [21]. Elevated hyaluronidase expression was accompanied by the accumulation of low molecular mass (3–10 disaccharides) HA, suggesting that increased hyaluronidase expression within ischemic CNS lesions is accompanied by the accumulation of HA digestion products.

Hyal1 mRNA was also elevated in rats following a craniotomy or a controlled cortical impact injury [30]. Other hyaluronidases, including PH20/SPAM1, were also detected and showed trends of increased expression but the changes were not significant. Similarly, hyaluronidases were elevated in demyelinating lesions where HA accumulates. Sloane and coworkers [32] found that OPCs express multiple hyaluronidases including PH20/SPAM1 (referred to henceforth as PH20) by immunocytochemistry. This result was surprising, since previous studies had not detected PH20 in the brain. A subsequent study demonstrated that PH20 is expressed by mouse astrocytes and OPCs both* in vitro* and in demyelinating mouse and human lesions by immunohistochemistry and, in mice, by RT-PCR [55]. This amplified RT-PCR product was the same size as PH20 amplified from mouse testes, did not originate from genomic DNA, and was confirmed to be PH20 by sequencing. Furthermore, infecting OPCs with a lentivirus carrying the cDNA for PH20, but not for other hyaluronidases normally expressed by OPCs, blocked OPC differentiation [55]. Treatment of OPCs* in vitro* with a broad-spectrum hyaluronidase inhibitor (ascorbate 6-hexadecanoate) promoted OPC maturation [32, 55] while treatment of demyelinating lesions with the inhibitor overcame the inhibitory effects of high molecular weight HA on remyelination, leading to functional recovery [55].

Further evidence that PH20 is elevated in the CNS following injury comes from a sheep model of prenatal white matter injury described above, where elevated ovine PH20 expression was confirmed through multiple approaches [24]. A single PH20 transcript was detected using a nested RT-PCR assay of mRNAs isolated from control and injured white matter in two separate labs. This transcript was the same size as in fetal ovine testes, was obtained with multiple primer sets, did not originate from genomic DNA, and was confirmed by sequencing to be PH20. PH20 was also detected in fetal ovine white matter by RNA-Seq in data from 10 animals, with the fetal ovine PH20 sequence confirmed to be highly homologous to rodent and human PH20. Finally, PH20 was detected by immunohistochemistry in fetal sheep brain tissues using two separate polyclonal antisera from rabbit and chicken. Lower levels of PH20 staining in controls relative to the white matter injury groups support the notion that PH20 expression is elevated following prenatal hypoxic-ischemic injury. All together, these data support the hypothesis that PH20 and possibly other hyaluronidases block OPC maturation and remyelination.

### 4.2. Hyaluronidases Can Influence Neuronal Activity

It is unclear if endogenous hyaluronidases influence neuronal function. However, a number of studies have suggested that HA within perineuronal nets can influence neuron activity that can be altered by hyaluronidase treatment. For example, the removal of perineuronal nets using hyaluronidase in hippocampal slices from young rats reduced the width of the synaptic cleft and increased the amplitude of excitatory postsynaptic potentials in CA1 axodendritic connections [56]. From this and other studies, it is reasonable to conclude that HA-rich perineuronal nets could influence both the efficacy and architecture of synapses. Furthermore, the controlled degradation of HA in perineuronal nets may be a mechanism used to alter synaptic plasticity underlying learning and memory. Hyaluronidase has also been reported to regulate *α*-amino-3-hydroxy-5-methyl-4-isoxazolepropionic acid (AMPA) receptor trafficking into and out of the synapse [57] suggesting that HA may control the plasticity of individual synapses by creating distinct membrane compartments and controlling the passive diffusion of molecules at the cell surface. Interestingly, perisynaptic HA may influence lateral mobility at the plasma membrane differently on spiny versus aspiny neurons [58]. How this perineuronal HA is regulated is unclear. It is intriguing to speculate, however, that hyaluronidases expressed by neurons or local glial cells may regulate neuronal activity through the regulated catabolism of HA.

### 4.3. HA Digestion Products Generated by Hyaluronidases Can Influence Neural Progenitor Cell Behaviors

While the digestion of HA by hyaluronidases may relieve signaling induced by high molecular weight HA, HA digestion products generated by hyaluronidases may also have their own distinct biological activities in the CNS. For example, digestion products of HA generated by bovine testicular hyaluronidase (whose activity is mostly PH20), but not those of another hyaluronidase directly blocked OPC differentiation and remyelination [55]. This finding is interesting in light of the observation that HA digestion products of sizes that are consistent with those predicted to be generated by hyaluronidase activity accumulate in demyelinated multiple sclerosis lesions [32]. It remains to be determined whether PH20 digestion products directly impact OPC maturation in ischemic CNS injuries the way they do in adult demyelinating lesions.

Additional evidence supporting the possibility that hyaluronidase-generated HA products could influence nervous system repair comes from studies utilizing HA oligosaccharides. For example, an HA tetrasaccharide was reported to improve functional recovery following spinal cord injury, possibly by inducing the expression of brain-derived neurotrophic factor (BDNF) and vascular endothelial growth factor (VEGF) in astrocytes within the injury environment [59]. HA tetrasaccharides can also promote axonal outgrowth and promote peripheral nerve regeneration [60]. It is unclear if these tetrasaccharides are blocking interactions between other sizes of HA and their receptors, or if they are mimicking the effects of HA digestion products.

These studies indicate that high molecular weight and digested forms of HA each have distinct functions following nervous system injury and during recovery. Certain sizes of HA products that accumulate in the damaged CNS may induce cellular signals that prevent differentiation and inhibit repair, while others may induce distinct signals that support these activities. Identifying how these products are generated, the precise sizes of HA fragments that influence cell signaling in neural progenitor populations, and the receptors and downstream effectors that regulate these signals will likely lead to novel strategies to promote nervous system repair.

## 5. HA Receptors Are Elevated in the Damaged CNS

There are a number of receptors that signal in response to HA [61]. Among these receptors, CD44, the receptor for HA-mediated motility (RHAMM), Stabilin-2 (the HA receptor for endocytosis), lymphatic vessel endothelial hyaluronan receptor-1 (LYVE-1), and HA-binding Toll-Like Receptors (TLR-2 and TLR-4) are expressed in either the normal or diseased CNS. The roles for most of these receptors in the brain and spinal cord are not clear. However, several studies have implicated CD44 in nervous system development, homeostasis, repair, and injury responses.

### 5.1. CD44 Is Elevated following CNS Insults and Is Required for CNS Development

HA binds to CD44 via an extracellular domain that shares homology with cartilage link protein. Multiple forms of CD44 are generated as a result of extensive RNA splicing and posttranslational modifications [62], both of which can alter the binding affinity of HA to CD44. In general, CD44 preferentially binds high molecular weight forms of HA, although lower MW forms of HA may also signal via CD44. CD44 has been shown to influence multiple cellular behaviors including survival, proliferation, migration, and differentiation via interactions with a variety of downstream intracellular signaling molecules. These functions are linked to CD44-mediated signaling through the CD44 cytoplasmic tail, which interacts with a number of intracellular proteins including Src family kinases and members of the ezrin, radixin, moesin family of actin-associated proteins, including the merlin tumor suppressor protein [62].

CD44 is expressed throughout both the central and peripheral nervous systems predominantly by glial cells, although some neuronal populations are at least transiently CD44-positive [5]. In parallel with HA, CD44 expression increases in the injured CNS coincident with gliosis [63]. In traumatic CNS injuries, for example, CD44 is chronically elevated within areas of reactive gliosis [64, 65]. CD44 is also elevated following ischemia in the brains of adult animals and humans [19, 21, 66, 67] and may influence inflammatory responses following ischemic brain injury [68]. CD44 is similarly elevated in lesions from patients [23] and sheep [24] with perinatal hypoxic-ischemic cerebral white matter injuries, in the gray matter of aged macaques [36], and in patients with evidence of vascular brain injury associated with age-related cognitive decline [39]. Finally, CD44 expression is increased in a mouse model of amyotrophic lateral sclerosis (ALS; [69]). In each of these conditions, CD44 localized to reactive astrocytes or microglia.

Demyelinating lesions demonstrate highly elevated CD44 expression on reactive astrocytes and OPCs in conjunction with elevated HA [31, 63, 70]. Transgenic elevation of CD44 in OPCs leads to HA accumulation and the persistence of OPCs that fail to differentiate into OLs [71]. These mice are phenotypically similar to* shiverer* mice that lack CNS myelin. Furthermore, chronic elevation of CD44 on OPCs leads to pericellular HA accumulation [31]. Thus, chronically elevated CD44 can contribute to HA accumulation that contributes to the failure of OPC maturation. It remains unclear if CD44 is required for the effects of HA digestion products on OPC maturation and remyelination. In contrast, loss of CD44 leads to impaired OPC migration into demyelinating lesions [50] suggesting that CD44 may be required for OPC recruitment following CNS insults. Together, these findings suggest that CD44 expression must be tightly regulated in OPCs to ensure that they effectively migrate to CNS lesions and differentiate into myelinating oligodendrocytes.

CD44 is also elevated in the hippocampus following seizures [25, 72]. Although the significance of this expression is unclear, mossy fiber sprouting can be inhibited using CD44 function-blocking antibodies [25]. Furthermore, CD44 null mice demonstrate hippocampal learning and memory deficits, consistent with abnormalities in adult hippocampal function [73]. These data suggest that, similar to OPCs, CD44 expression by cells in the hippocampus must be tightly regulated to maintain hippocampal function.

### 5.2. RHAMM May Influence Axon Outgrowth and Glial Cell Motility

Like CD44, RHAMM also exists in multiple isoforms and is expressed by at least subsets of neurons and glial cells, especially oligodendrocytes [74]. Unlike CD44, RHAMM can function both in the cytoplasm and at the cell surface as a glycophosphatidylinositol-anchored protein. Intracellular RHAMM binds to a number of structures including microtubules, actin, the centrosome and the mitotic spindle, and podosomes. HA binding to RHAMM can promote cell migration and growth through interactions with a variety of intracellular signaling molecules such as focal adhesion kinase (FAK), by inducing changes in actin and microtubule dynamics [75]. Interestingly, RHAMM can also interact with CD44 to influence extracellular signal-regulated kinase (ERK) activity that is required to promote cell migration [76].

Like CD44, RHAMM is observed in astrocytes in peri-infarct areas following ischemia [20] and has been implicated in promoting axonal growth and astrocyte and microglial motility [77]. It is unclear, however, how RHAMM-HA interactions influence CNS injury outcomes. Interestingly, RHAMM can bind to the signaling mediator calmodulin in a calcium-dependent manner [78], suggesting a role for RHAMM in calmodulin-mediated cell signaling in the CNS.

### 5.3. Toll-Like Receptors May Regulate Responses to HA Digestion Products in the Injured CNS

A number of studies have suggested that HA is a ligand for Toll-Like Receptors (TLRs). Both TLR2 and TLR4 have been reported to bind HA [79] and their expression has been detected in NSPCs [80] where they may play a role in regulating neurogenesis. TLRs are also expressed by microglia and astrocytes [81]. Sloane and coworkers [32] have reported that TLR2 is expressed by OPCs and that hyaluronidase-generated HA digestion products signal through TLR2 to inhibit OPC maturation, although roles for additional receptors in OPC responses to HA products have not been ruled out.

Altogether, studies to date support the notion that multiple HA receptors are involved in nervous system responses to injury, regulating cell migration, axon outgrowth, and differentiation. What remains to be determined is how these receptors interact to influence each of these cellular behaviors, how receptor activation influences specific intracellular signaling cascades, and which receptors respond to high molecular weight HA versus HA digestion products generated by hyaluronidases.

## 6. Conclusions

**Figure 2:**
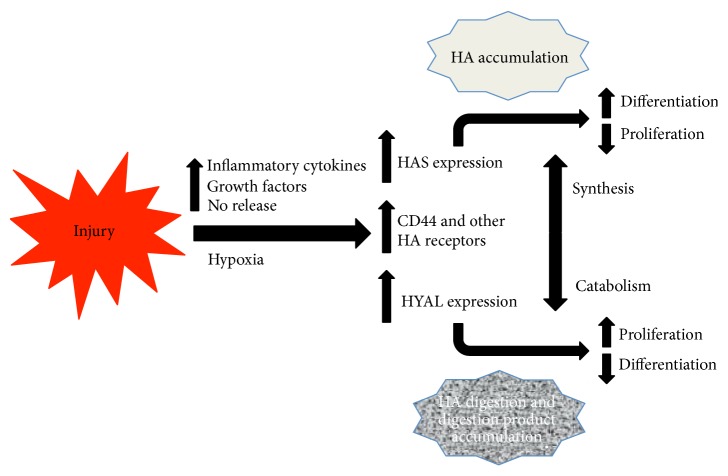
HA synthases, HA receptors, and hyaluronidases are transcriptionally upregulated in response to injury by inflammatory mediators and other injury-induced mediators. As a result, HA increases in injured tissues but also is digested. The balance between HA synthesis and catabolism influences cellular behaviors, such as proliferation and differentiation, either by influencing signals induced by high molecular weight HA or due to the accumulation of HA digestion products that have their own biological activities.

**Figure 3:**
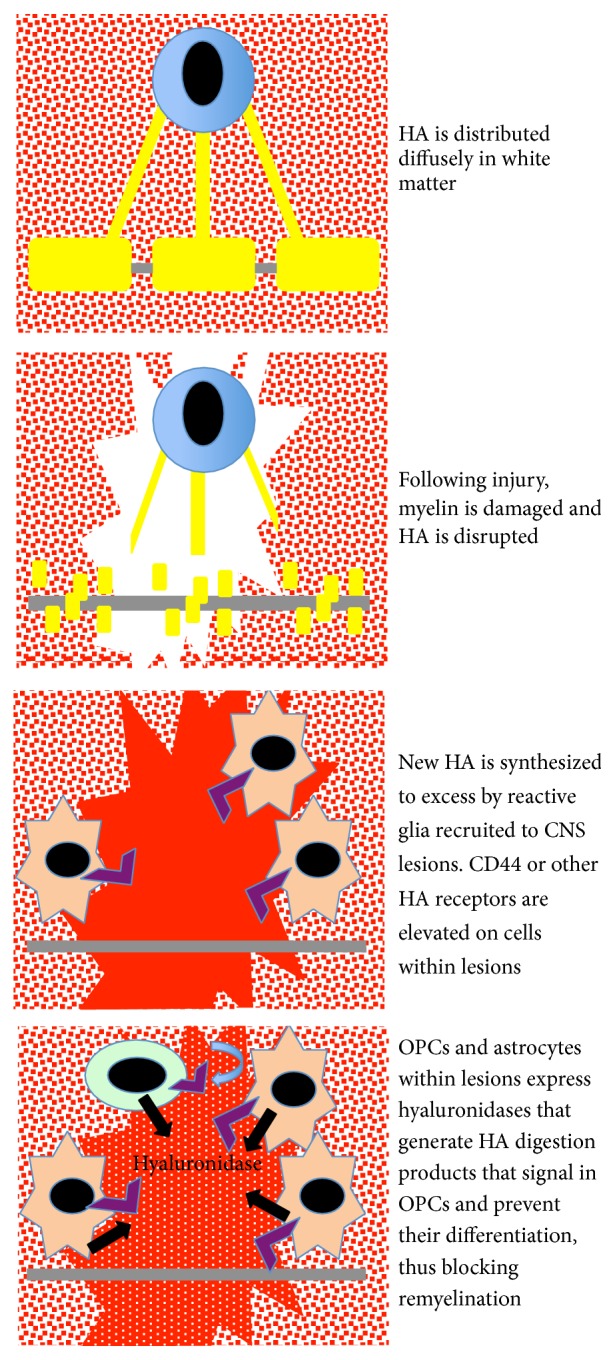
A single OL (blue cell) can form myelin (yellow) for multiple internodes of the same axon (gray) or for many axons. In uninjured white matter, HA (red) is diffuse while in perineuronal nets HA is at much higher density (not shown). Following injury, myelin and oligodendrocytes are destroyed and HA is initially disrupted. HA later accumulates at higher than normal levels coincident with the appearance of reactive astrocytes (orange cells). CD44 and possibly other HA receptors (purple) are elevated on astrocytes and OPCs recruited to lesions. Both astrocytes and recruited OPCs (green cell) then express hyaluronidases (including PH20; black arrows in lower panel) that digest the excess HA within lesions. The resulting HA digestion products that accumulate in the injury microenvironment feed back on OPCs (blue arrow in lower panel) and prevent their differentiation and subsequent remyelination.

HA synthesis and catabolism are both induced following insults to various tissues, including the CNS. Following most if not all forms of CNS injury, the HA-based ECM is initially disrupted, leading to the loss of HA within new lesions. However, soon after the initial CNS insult, HA accumulates predominantly through transcriptional upregulation of* HAS* genes by astrocytes and other reactive glia. HA accumulation is accompanied by transcriptional upregulation of CD44 and possibly other transmembrane HA receptors leading to both enhanced HA signaling and anchoring of cell surface HA, contributing to further HA accumulation in the injury microenvironment. Such anchoring of HA to the cell surface by CD44 has been described in activated brain endothelial cells [82]. In conjunction with the transcriptional activation of* HAS* genes and HA receptors, the expression of one or more hyaluronidases is also induced, leading to the digestion and clearance of the accumulated HA in the lesions. The balance between HA accumulation and HA degradation by hyaluronidases can influence a number of cell behaviors, such as proliferation and differentiation in NSPC niches (Figure 2). Interestingly, HAS, HA receptor, and hyaluronidase expression may each be influenced by the same milieu of proinflammatory mediators that are induced following tissue damage. Disruption in the balance between HA synthesis and catabolism can lead to either excess high molecular weight HA accumulation or the accumulation of HA digestion products that can negatively impact CNS repair. The best example of this latter outcome has been demonstrated in the case of remyelination failure, where the accumulation of HA digestion products inhibits OPC maturation in demyelinating lesions (Figure 3).

In CNS diseases and injuries, it will be important to determine to what degree the disruption of high molecular weight HA versus the accumulation of HA digestion products is important for recovery. For example, following seizures, it is conceivable that maintaining the HA matrix is important to protect perineuronal nets and possibly NSPC survival and differentiation in NSPC niches. This could be achieved by blocking hyaluronidase activity or expression if hyaluronidases are involved in HA degradation within NSPC niches or by elevating HAS activity. Inhibiting hyaluronidase activity has been effective in other biological systems, including blocking tumor growth in at least some cancer cells (e.g., [83]) and to promote OPC maturation [55]. Blocking the activation of receptors that respond to HA digestion products may also be an efficacious strategy to promote OPC maturation and myelination following perinatal hypoxia-ischemia and in demyelinating diseases. As the contributions of HA synthesis, catabolism and signaling become more clear in different types of CNS insults, we will gain new clues about how targeting HA synthases, hyaluronidases, and HA receptors could lead to novel therapies to promote CNS repair.

## References

[1] Bignami A., Hosley M., Dahl D. (1993). Hyaluronic acid and hyaluronic acid-binding proteins in brain extracellular matrix. *Anatomy and Embryology*.

[2] Bandtlow C. E., Zimmermann D. R. (2000). Proteoglycans in the developing brain: new conceptual insights for old proteins. *Physiological Reviews*.

[3] Dityatev A., Schachner M. (2003). Extracellular matrix molecules and synaptic plasticity. *Nature Reviews Neuroscience*.

[4] Sherman L. S., Back S. A. (2008). A ‘GAG’ reflex prevents repair of the damaged CNS. *Trends in Neurosciences*.

[5] Preston M., Sherman L. S. (2011). Neural stem cell niches: roles for the hyaluronan-based extracellular matrix. *Frontiers in Bioscience*.

[6] Jakeman L. B., Williams K. E., Brautigam B. (2014). In the presence of danger: the extracellular matrix defensive response to central nervous system injury. *Neural Regeneration Research*.

[7] Levy A. D., Omar M. H., Koleske A. J. (2014). Extracellular matrix control of dendritic spine and synapse structure and plasticity in adulthood. *Frontiers in Neuroanatomy*.

[8] Lau L. W., Cua R., Keough M. B., Haylock-Jacobs S., Yong V. W. (2013). Pathophysiology of the brain extracellular matrix: a new target for remyelination. *Nature Reviews Neuroscience*.

[9] Kwok J. C. F., Dick G., Wang D., Fawcett J. W. (2011). Extracellular matrix and perineuronal nets in CNS repair. *Developmental Neurobiology*.

[10] Härtig W., Derouiche A., Welt K. (1999). Cortical neurons immunoreactive for the potassium channel Kv3.1b subunit are predominantly surrounded by perineuronal nets presumed as a buffering system for cations. *Brain Research*.

[11] Cregg J. M., DePaul M. A., Filous A. R., Lang B. T., Tran A., Silver J. (2014). Functional regeneration beyond the glial scar. *Experimental Neurology*.

[12] Gaudet A. D., Popovich P. G. (2014). Extracellular matrix regulation of inflammation in the healthy and injured spinal cord. *Experimental Neurology*.

[13] Burnside E. R., Bradbury E. J. (2014). Review: manipulating the extracellular matrix and its role in brain and spinal cord plasticity and repair. *Neuropathology and Applied Neurobiology*.

[14] Polansky J. R., Toole B. P., Gross J. (1974). Brain hyaluronidase: changes in activity during chick development. *Science*.

[15] Sampaio L. O., Dietrich C. P. (1981). Changes of sulfated mucopolysaccharides and mucopolysaccharidases during fetal development. *The Journal of Biological Chemistry*.

[16] Shibata S., Cho K. H., Kim J. H., Abe H., Murakami G., Cho B. H. (2013). Expression of hyaluronan (hyaluronic acid) in the developing laminar architecture of the human fetal brain. *Annals of Anatomy*.

[17] Arranz A. M., Perkins K. L., Irie F. (2014). Hyaluronan deficiency due to Has3 knock-out causes altered neuronal activity and seizures via reduction in brain extracellular space. *Journal of Neuroscience*.

[18] Laurent U. B. G., Laurent T. C., Hellsing L. K., Persson L., Hartman M., Lilja K. (1996). Hyaluronan in human cerebrospinal fluid. *Acta Neurologica Scandinavica*.

[19] Wang H., Zhan Y., Xu L., Feuerstein G. Z., Wang X. (2001). Use of suppression subtractive hybridization for differential gene expression in stroke: discovery of CD44 gene expression and localization in permanent focal stroke in rats. *Stroke*.

[20] Lindwall C., Olsson M., Osman A. M., Kuhn H. G., Curtis M. A. (2013). Selective expression of hyaluronan and receptor for hyaluronan mediated motility (Rhamm) in the adult mouse subventricular zone and rostral migratory stream and in ischemic cortex. *Brain Research*.

[21] Al'Qteishat A., Gaffney J., Krupinski J. (2006). Changes in hyaluronan production and metabolism following ischaemic stroke in man. *Brain*.

[22] Tang S., Yeh S., Tsai L. (2014). Association between plasma levels of hyaluronic acid and functional outcome in acute stroke patients. *Journal of Neuroinflammation*.

[23] Buser J. R., Maire J., Riddle A. (2012). Arrested preoligodendrocyte maturation contributes to myelination failure in premature infants. *Annals of Neurology*.

[24] Hagen M. W., Riddle A., McClendon E. (2014). Role of recurrent hypoxia-ischemia in preterm white matter injury severity. *PLoS ONE*.

[25] Bausch S. B. (2006). Potential roles for hyaluronan and CD44 in kainic acid-induced mossy fiber sprouting in organotypic hippocampal slice cultures. *Neuroscience*.

[26] Mcrae P. A., Baranov E., Rogers S. L., Porter B. E. (2012). Persistent decrease in multiple components of the perineuronal net following status epilepticus. *European Journal of Neuroscience*.

[27] Perosa S. R., Porcionatto M. A., Cukiert A. (2002). Glycosaminoglycan levels and proteoglycan expression are altered in the hippocampus of patients with mesial temporal lobe epilepsy. *Brain Research Bulletin*.

[28] Mansour H., Asher R., Dahl D., Labkovsky B., Perides G., Bignami A. (1990). Permissive and non-permissive reactive astrocytes: Immunofluorescence study with antibodies to the glial hyaluronate-binding protein. *Journal of Neuroscience Research*.

[29] Struve J., Maher P. C., Li Y.-Q. (2005). Disruption of the hyaluronan-based extracellular matrix in spinal cord promotes astrocyte proliferation. *Glia*.

[30] Xing G., Ren M., Verma A. (2014). Divergent temporal expression of hyaluronan metabolizing enzymes and receptors with craniotomy vs. controlled-cortical impact injury in rat brain: a pilot study. *Frontiers in Neurology*.

[31] Back S. A., Tuohy T. M. F., Chen H. (2005). Hyaluronan accumulates in demyelinated lesions and inhibits oligodendrocyte progenitor maturation. *Nature Medicine*.

[32] Sloane J. A., Batt C., Ma Y., Harris Z. M., Trapp B., Vartanian T. (2010). Hyaluronan blocks oligodendrocyte progenitor maturation and remyelination through TLR2. *Proceedings of the National Academy of Sciences of the United States of America*.

[33] Chang A., Staugaitis S. M., Dutta R. (2012). Cortical remyelination: a new target for repair therapies in multiple sclerosis. *Annals of Neurology*.

[34] Bugiani M., Postma N., Polder E. (2013). Hyaluronan accumulation and arrested oligodendrocyte progenitor maturation in vanishing white matter disease. *Brain*.

[35] Jenkins H. G., Bachelard H. S. (1988). Developmental and age-related changes in rat brain glycosaminoglycans. *Journal of Neurochemistry*.

[36] Cargill R., Kohama S. G., Struve J. (2012). Astrocytes in aged nonhuman primate brain gray matter synthesize excess hyaluronan. *Neurobiology of Aging*.

[37] Jenkins H. G., Bachelard H. S. (1988). Glycosaminoglycans in cortical autopsy samples from Alzheimer brain. *Journal of Neurochemistry*.

[38] Suzuki K., Katzman R., Korey S. R. (1965). Chemical studies on Alzheimer’s disease. *Journal of Neuropathology and Experimental Neurology*.

[39] Back S. A., Kroenke C. D., Sherman L. S. (2011). White matter lesions defined by diffusion tensor imaging in older adults. *Annals of Neurology*.

[40] Nägga K., Hansson O., van Westen D., Minthon L., Wennström M. (2014). Increased levels of hyaluronic acid in cerebrospinal fluid in patients with vascular dementia. *Journal of Alzheimer's Disease*.

[41] Franklin R. J., Ffrench-Constant C. (2008). Remyelination in the CNS: from biology to therapy. *Nature Reviews Neuroscience*.

[42] Wolswijk G. (1998). Chronic stage multiple sclerosis lesions contain a relatively quiescent population of oligodendrocyte precursor cells. *Journal of Neuroscience*.

[43] Scolding N., Franklin R., Stevens S., Heldin C.-H., Compston A., Newcombe J. (1998). Oligodendrocyte progenitors are present in the normal adult human CNS and in the lesions of multiple sclerosis. *Brain*.

[44] Chang A., Nishiyama A., Peterson J., Prineas J., Trapp B. D. (2000). NG2-positive oligodendrocyte progenitor cells in adult human brain and multiple sclerosis lesions. *Journal of Neuroscience*.

[45] Maeda Y., Solanky M., Menonna J., Chapin J., Li W., Dowling P. (2001). Platelet-derived growth factor-alpha receptor-positive oligodendroglia are frequent in multiple sclerosis lesions. *Annals of Neurology*.

[46] Chang A., Tourtellotte W. W., Rudick R., Trapp B. D. (2002). Premyelinating oligodendrocytes in chronic lesions of multiple sclerosis. *The New England Journal of Medicine*.

[47] Wolswijk G. (2002). Oligodendrocyte precursor cells in the demyelinated multiple sclerosis spinal cord. *Brain*.

[48] Dean J. M., Riddle A., Maire J. (2011). An organotypic slice culture model of chronic white matter injury with maturation arrest of oligodendrocyte progenitors. *Molecular Neurodegeneration*.

[49] Lin C.-M., Lin J.-W., Chen Y.-C. (2009). Hyaluronic acid inhibits the glial scar formation after brain damage with tissue loss in rats. *Surgical Neurology*.

[50] Piao J.-H., Wang Y., Duncan I. D. (2013). CD44 is required for the migration of transplanted oligodendrocyte progenitor cells to focal inflammatory demyelinating lesions in the spinal cord. *Glia*.

[51] Fuxe K., Tinner B., Chadi G., Härfstrand A., Agnati L. F. (1994). Evidence for a regional distribution of hyaluronic acid in the rat brain using a highly specific hyaluronic acid recognizing protein. *Neuroscience Letters*.

[52] Solis M. A., Chen Y.-H., Wong T. Y., Bittencourt V. Z., Lin Y.-C., Huang L. L. H. (2012). Hyaluronan regulates cell behavior: a potential niche matrix for stem cells. *Biochemistry Research International*.

[53] Liang Y., Walczak P., Bulte J. W. M. (2013). The survival of engrafted neural stem cells within hyaluronic acid hydrogels. *Biomaterials*.

[54] Zhong J., Chan A., Morad L., Kornblum H. I., Carmichael S. T. (2010). Hydrogel matrix to support stem cell survival after brain transplantation in stroke. *Neurorehabilitation and Neural Repair*.

[55] Preston M., Gong X., Su W. (2013). Digestion products of the PH20 hyaluronidase inhibit remyelination. *Annals of Neurology*.

[56] Kul'chitskii S. V., Yakubovich N. V., Emel'yanova A. A., Garkun Y. S., Pashkevich S. G., Kul'chitskii V. A. (2009). Changes in neuropil ultrastructure in hippocampal field CA1 in rat pups after application of hyaluronidase. *Neuroscience and Behavioral Physiology*.

[57] Frischknecht R., Heine M., Perrais D., Seidenbecher C. I., Choquet D., Gundelfinger E. D. (2009). Brain extracellular matrix affects AMPA receptor lateral mobility and short-term synaptic plasticity. *Nature Neuroscience*.

[58] Klueva J., Gundelfinger E. D., Frischknecht R. R., Heine M. (2014). Intracellular Ca^2+^ and not the extracellular matrix determines surface dynamics of AMPA-type glutamate receptors on aspiny neurons. *Philosophical Transactions of the Royal Society of London B: Biological Science*.

[59] Wang J., Wang X., Wei J., Wang M. (2015). Hyaluronan tetrasaccharide exerts neuroprotective effect and promotes functional recovery after acute spinal cord injury in rats. *Neurochemical Research*.

[60] Torigoe K., Tanaka H. F., Ohkochi H. (2011). Hyaluronan tetrasaccharide promotes regeneration of peripheral nerve: in vivo analysis by film model method. *Brain Research*.

[61] Vigetti D., Karousou E., Viola M., Deleonibus S., De Luca G., Passi A. (2014). Hyaluronan: biosynthesis and signaling. *Biochimica et Biophysica Acta—General Subjects*.

[62] Ponta H., Sherman L., Herrlich P. A. (2003). CD44: from adhesion molecules to signalling regulators. *Nature Reviews Molecular Cell Biology*.

[63] Vogel H., Butcher E. C., Picker L. J. (1992). H-CAM expression in the human nervous system: evidence for a role in diverse glial interactions. *Journal of Neurocytology*.

[64] Stylli S. S., Kaye A. H., Novak U. (2000). Induction of CD44 expression in stab wounds of the brain: long term persistence of CD44 expression. *Journal of Clinical Neuroscience*.

[65] Jones L. L., Liu Z., Shen J., Werner A., Kreutzberg G. W., Raivich G. (2000). Regulation of the cell adhesion molecule CD44 after nerve transection and direct trauma to the mouse brain. *Journal of Comparative Neurology*.

[66] Al Qteishat A., Gaffney J. J., Krupinski J., Slevin M. (2006). Hyaluronan expression following middle cerebral artery occlusion in the rat. *NeuroReport*.

[67] Kang W.-S., Choi J.-S., Shin Y.-J. (2008). Differential regulation of osteopontin receptors, CD44 and the *α*
_v_ and *β*
_3_ integrin subunits, in the rat hippocampus following transient forebrain ischemia. *Brain Research*.

[68] Wang X., Xu L., Wang H., Zhan Y., Puré E., Feuerstein G. Z. (2002). CD44 deficiency in mice protects brain from cerebral ischemia injury. *Journal of Neurochemistry*.

[69] Matsumoto T., Imagama S., Hirano K. (2012). CD44 expression in astrocytes and microglia is associated with ALS progression in a mouse model. *Neuroscience Letters*.

[70] Alldinger S., Fonfara S., Kremmer E., Baumgärtner W. (2000). Up-regulation of the hyaluronate receptor CD44 in canine distemper demyelinated plaques. *Acta Neuropathologica*.

[71] Tuohy T. M. F., Wallingford N., Liu Y. (2004). CD44 overexpression by oligodendrocytes: a novel mouse model of inflammation-independent demyelination and dysmyelination. *Glia*.

[72] Borges K., McDermott D. L., Dingledine R. (2004). Reciprocal changes of CD44 and GAP-43 expression in the dentate gyrus inner molecular layer after status epilepticus in mice. *Experimental Neurology*.

[73] Raber J., Olsen R. H. J., Su W. (2014). CD44 is required for spatial memory retention and sensorimotor functions. *Behavioral Brain Research*.

[74] Lynn B. D., Li X., Cattini P. A., Turley E. A., Nagy J. I. (2001). Identification of sequence, protein isoforms, and distribution of the hyaluronan-binding protein RHAMM in adult and developing rat brain. *Journal of Comparative Neurology*.

[75] Hall C. L., Wang C., Lange L. A., Turley E. A. (1994). Hyaluronan and the hyaluronan receptor RHAMM promote focal adhesion turnover and transient tyrosine kinase activity. *The Journal of Cell Biology*.

[76] Hamilton S. R., Fard S. F., Paiwand F. F. (2007). The hyaluronan receptors CD44 and Rhamm (CD168) form complexes with ERK1,2 that sustain high basal motility in breast cancer cells. *The Journal of Biological Chemistry*.

[77] Turley E. A., Hossain M. Z., Sorokan T., Jordan L. M., Nagy J. I. (1994). Astrocyte and microglial motility in vitro is functionally dependent on the hyaluronan receptor RHAMM. *Glia*.

[78] Lynn B. D., Turley E. A., Nagy J. I. (2001). Subcellular distribution, calmodulin interaction, and mitochondrial association of the hyaluronan-binding protein RHAMM in rat brain. *Journal of Neuroscience Research*.

[79] Sloane J. A., Blitz D., Margolin Z., Vartanian T. (2010). A clear and present danger: endogenous ligands of Toll-like receptors. *NeuroMolecular Medicine*.

[80] Rolls A., Shechter R., London A. (2007). Toll-like receptors modulate adult hippocampal neurogenesis. *Nature Cell Biology*.

[81] Gurley C., Nichols J., Liu S., Phulwani N. K., Esen N., Kielian T. (2008). Microglia and astrocyte activation by toll-like receptor ligands: modulation by PPAR-gamma agonists. *PPAR Research*.

[82] Winkler C. W., Foster S. C., Matsumoto S. G. (2012). Hyaluronan anchored to activated CD44 on central nervous system vascular endothelial cells promotes lymphocyte extravasation in experimental autoimmune encephalomyelitis. *Journal of Biological Chemistry*.

[83] Benitez A., Yates T. J., Lopez L. E., Cerwinka W. H., Bakkar A., Lokeshwar V. B. (2011). Targeting hyaluronidase for cancer therapy: antitumor activity of sulfated hyaluronic acid in prostate cancer cells. *Cancer Research*.

